# Interaction Mechanisms Between Major Depressive Disorder and Non-alcoholic Fatty Liver Disease

**DOI:** 10.3389/fpsyt.2021.711835

**Published:** 2021-12-13

**Authors:** Qi Shao, Yiping Wu, Jing Ji, Tian Xu, Qiaoyu Yu, Chongyang Ma, Xuejing Liao, Fafeng Cheng, Xueqian Wang

**Affiliations:** College of Traditional Chinese Medicine, Beijing University of Chinese Medicine, Beijing, China

**Keywords:** chronic stress, MD, NAFLD, GC, inflammatory factors, gut permeability, TH

## Abstract

Major depressive disorder (MDD), which is highly associated with non-alcoholic fatty liver disease (NAFLD), has complex pathogenic mechanisms. However, a limited number of studies have evaluated the mutual pathomechanisms involved in MDD and NAFLD development. Chronic stress-mediated elevations in glucocorticoid (GC) levels play an important role in the development of MDD-related NAFLD. Elevated GC levels can induce the release of inflammatory factors and changes in gut permeability. Elevated levels of inflammatory factors activate the hypothalamic–pituitary–adrenal (HPA) axis, which further increases the release of GC. At the same time, changes in gut permeability promote the release of inflammatory factors, which results in a vicious circle among the three, causing disease outbreaks. Even though the specific role of the thyroid hormone (TH) in this pathogenesis has not been fully established, it is highly correlated with MDD and NAFLD. Therefore, changing lifestyles and reducing psychological stress levels are necessary measures for preventing MDD-related NAFLD. Among them, GC inhibitors and receptor antagonists may be key in the alleviation of early and mid-term disease progression. However, combination medications may be important in late-stage diseases, but they are associated with various side effects. Traditional Chinese medicines have been shown to be potential therapeutic alternatives for such complex diseases.

## Introduction

Major depressive disorder (MDD) is characterized by loss of interest, difficulty in paying attention, decreased appetite, and suicidal ideation, among other abnormal cognitive, behavioral, and social functions, with low mood as the main symptom (1). Chronic stress is highly correlated with the onset of MDD (2). With societal developments and acceleration of the pace of life, stress due to various life pressures has significantly increased, leading to annually increasing MDD incidences. It is projected that by 2030, MDD will be the leading cause of the global disease burden (3). Long-term psychological stress plays an important role in initiating and mediating the occurrence and development of many major diseases (4–6). Non-alcoholic fatty liver (NAFL) disease (NAFLD), a clinicopathologic syndrome, is characterized by diffuse hepatic alveolar steatosis and fat storage in liver lobules, with the exception of alcohol and other definite liver-damaging factors, including NAFL and non-alcoholic steatohepatitis (NASH) (7, 8). However, the natural history of this disorder is unknown (9, 10). Studies have shown that NAFLD has the risk of progression to cirrhosis and cancer (11, 12). Not only is NAFLD the world's most common chronic liver disease, but it has also become a common cause of other liver diseases (8). Unfortunately, NAFLD and MDD mediate and promote the progression of each other (13, 14). Interactions between these diseases increase the complexity of pathomechanisms involved in the pathogenesis of these two kinds of diseases. By analyzing and summarizing social, clinical, and scientific literature, we elucidate on the relationship between MDD and NAFLD as well as the potential common pathogenic mechanisms. This review provides a basis for clinical prevention and treatment of NAFLD.

## Correlations Between Major Depressive Disorder and Non-alcoholic Fatty Liver Disease

### Depressive States Mediate Non-alcoholic Fatty Liver Disease Occurrence and Development

In a large study of relationships between psychological stress and NAFLD, elevated stress levels were associated with a high prevalence of NAFLD (15). This finding was confirmed in an assessment of U.S. adults from 2007 to 2016 by Kim et al., who found that MDD patients were 1.6 to 2.2 times more likely to have NAFLD, relative to those without MDD (16). In a study involving 567 biopsy-confirmed NAFLD patients, after adjusting for potential confounding factors, Youssef et al. found that there was a dose-dependent relationship between the severity of depressive symptoms and the degree of hepatocyte swelling. Compared with non-depressed patients, subclinical MDD patients were 2.1 times more likely to exhibit hepatocyte swelling, while clinical MDD patients were 3.6 times more likely (17). Meanwhile, mentally and intellectually impaired youth are at a greater risk of liver steatosis development (18), and more significantly, there is a significant correlation between psychological distress and liver disease-associated mortality. In their survey of 166,631 people, Russ et al. found that the risk of liver disease-associated mortality significantly increased with increasing General Health Questionnaire (GHQ) (a health questionnaire consisting of 12 items) score, and they divided the GHQ score into 0 (no pain), 1–3, 4–6, and 7–12 points. After adjustment for age and gender, the risk ratio for the 7–12 group compared with the 0 group was 3.48, while after adjustment of health behaviors, socioeconomic conditions, and so on, the hazard ratio reduced to 2.59 (19). Due to the complexities of human disease mechanisms and other uncontrollable factors, the processes involved in depression-induced NAFLD occurrence and development should be evaluated in animal experiments. Donkelaar et al. orally administered corticosterone in mice for a long time to simulate a physiological stress state. They found that the mice gained weight, their insulin sensitivity was decreased, and they also exhibited depressive-like behaviors (20). Liu et al. established chronic stress models by subjecting mice to electric foot shock and restraint stress. They found that chronic stress caused increased triglyceride (TG) and total cholesterol (TC) levels while decreasing body weight, visceral fat mass, microvesicular steatosis, lobular inflammation, and ballooning degeneration (21). These studies show that depression plays an important role in NAFLD occurrence and development.

### Non-alcoholic Fatty Liver Disease Aggravates Depression

Depressive states mediate NAFLD occurrence and development, while NAFLD aggravates depressive states. For example, in a study of a large primary care team in Germany, Labenz et al. found that NAFLD patients were more likely to suffer from MDD than the control group without NAFLD. Therefore, they considered NAFLD to be an independent risk factor for MDD and anxiety (22). Weinstein et al. reported that NAFLD patients had a significantly higher prevalence of depressive disorders (27.2%) than controls (2%−5%) (23). These findings have been validated by many studies. Filipović et al. found that compared with those of healthy patients, NAFLD patients' volumes of the brain, white, and gray matter reduced while lateral ventricles increased. Moreover, the risk of cognitive decline, cognitive impairment, and cognitive deficits was four times higher in patients without NAFLD (24). In their assessment of the relationship between MDDs, generalized anxiety disorders (GADs), and NASH, Elwing et al. found that MDD and GADs were more highly expressed in NASH patients than in the control group. Moreover, the degree of steatosis in MDD patients and inflammation grade as well as fibrosis stage in GAD patients were higher than those of patients without a diagnosis of mental illness (13). The hypothesis that NAFLD aggravates depression has been verified in animal experiments. To elucidate on the impact of liver disease on MDD, Higarza et al. conducted preclinical evaluations in NASH animal models to verify whether there were behavioral and emotional changes. One of the studies used a forced swimming test to evaluate depression-like rat behaviors; they found that NASH rat models exhibited a lack of escape struggle compared with the normal group, implying that NASH induces neurobehavioral dysfunctions (25).

## Mutual Mediation Mechanisms Between Major Depressive Disorder and Non-alcoholic Fatty Liver Disease

### Elevated Glucocorticoid Levels

The hypothalamic–pituitary–adrenal (HPA) axis is a neuroendocrine axis composed of the hypothalamus, pituitary gland, and adrenal gland. It plays a key role in regulating the response of organisms to environmental changes (26). Stress impulses are transmitted from the nerves to the brain and then to the hypothalamus of the central nervous system (CNS). They mainly act on the corticotropin-releasing factor (CRF) in the paraventricular nucleus (PVN) of the hypothalamus. CRF is synthesized by neurons dividing small cells in PVN (27). As the main neurohormone that regulates the HPA axis, it plays an important role in psychological stress (26). Synthetic CRF enters the anterior pituitary through the pituitary portal system (28), whereby it binds the CRF1 receptor and initiates signal transduction of protein kinase A (PKA) and protein kinase C (PKC) to promote the synthesis and secretion of the adrenocorticotropic hormone (ACTH) (26). ACTH stimulates the adrenal cortex to secrete cortisol and release it into the blood (28). Under normal physiological conditions, cortisol acts on the hippocampus, PVN, and anterior pituitary gland; then, through a negative feedback loop mediated by the glucocorticoid (GC) receptor (GR), it inhibits the HPA axis, thereby suppressing the synthesis and secretion of CRF as well as ACTH (29). However, long-term psychological stress can lead to continuous hyperactivity of the HPA axis and increases GC levels (30, 31). Excess GC can damage cortisol receptors in the hippocampus at the high position of the HPA axis, causing the HPA axis to lose negative feedback regulation and, ultimately, leading to a hyperactivated HPA axis that leads to abnormally high GC levels (27).

#### Glucocorticoid and Major Depressive Disorder

Monoamine hypothesis, the most common hypothesis in pathophysiology, describes the molecular mechanisms of MDD. Most commonly used antidepressants have been developed according to the monoamine hypothesis. This hypothesis describes changes in 5-hydroxytryptamine (5-HT), norepinephrine (NE), and dopamine (DA) in the synaptic cleft (32). However, the current antidepressants developed on the basis of this hypothesis have not achieved the desired results (33). Therefore, the inflammatory mediator hypothesis (34) and a series of neural-related hypotheses, such as the neuroplasticity hypothesis/neurogenesis hypothesis (32), the neurotrophic factor (NF) hypothesis (35), the neuron regeneration hypothesis (36), and the neurosynaptic hypothesis, have been developed (37). These hypotheses describe the complexity and heterogeneity of MDD mechanisms. GC may be key to proving the existing types of hypotheses and an important target for MDD treatment (29, 38, 39).

GC exerts both anti-inflammatory and proinflammatory actions. Body responses to GC depend on GC concentrations, physiological states of the body's immune systems, and duration of stimulation (acute or chronic), among others (40). GC regulates interleukin-1β (IL-1β), interleukin-6 (IL-6), interleukin-6 (IL-8), nucleotide-binding oligomerization domain-like receptor protein 3 (NLRP3), tumor necrosis factor-α (TNF-α) and other inflammatory factors through the c-Jun N-terminal kinase (c-JNK), extracellular-regulated protein kinases (ERKs), phosphatidylinositol 3-kinase (PI3K), IκB kinase (IκK), nuclear factor kappa-B (NF-κB), and signal transducers and activators of transcription (STAT) pathways (41, 42). Meanwhile, elevated GC levels can suppress 5-HT synthesis by inhibiting the expressions of central tryptophan hydroxylase 2 (TPH2) (43) and can reduce NE synthesis (44). The HPA axis, GC, and hippocampal volumes have been shown to form a vicious circle in MDD patients: stress increases GC levels, and increased GC reduces the hippocampal volume by exerting negative effects on neuromorphologies (neuroplasticity hypothesis) and/or neurogenesis of dentate gyrus (neurogenesis hypothesis). The decrease in hippocampal volumes may lead to the failure of negative feedback of the HPA axis. Then, GC levels further increase, thereby aggravating the failure of the HPA axis, leading to MDD occurrence (32).

Second, GR can reduce the expressions of brain-derived NF (BDNF) by directly binding the restriction sequence upstream of exon IV (exon IV) (45). In addition, GC causes neuronal apoptosis and degeneration by affecting hippocampal neural stem cells (NSCs) and neural precursor cells (NPCs) (46). GC-regulated kinase 1 (SGK1) plays a key role in MDD. GC improves SGK1 activities by increasing the phosphorylation of SGK1 at serine 422 (Ser422) and threonine 256 (Thr256), leading to phosphorylation of GR at Ser203 and Ser211, thereby affecting its nuclear translocation and further leading to suppressed BDNF synthesis (33). In addition, SGK1 is involved in the regulation of the growth of dendrites (47), establishment of neuronal plasticity (48), and neuronal excitement (49), which may lead to MDD occurrence.

#### Glucocorticoid and Non-alcoholic Fatty Liver Disease

The pathogenesis of NAFLD has yet to be fully elucidated. The “second hit” theory is a classic theory to describe its pathogenesis (50). “The first hit” theory is centered on insulin resistance (IR), involving liver steatosis caused by the imbalance of fatty acid (FA) and TG metabolism in liver cells (51). “The second hit” theory is mainly centered on oxidative stress (OS), and it also involves liver inflammation, necrosis, and fibrosis that are a result of inflammatory cytokine production, lipid peroxidation, and mitochondrial dysfunction (52). Since the “second hit” theory does not fully explain NAFLD pathogenesis, studies have proposed the “multiple parallel hits” theory, which involves IR, lipotoxicity, inflammation, genetic polymorphism and epigenetics, adipokines, bile acid (BA), gut microbiota (GM), and environmental and dietary factors, among others (53). However, the “multiple parallel hits” theory cannot fully explain the interrelationship between complex mechanisms and is only considered to be a supplement to the original classic “second hit” theory. Among them, GC may be key in implementing these two theories (54).

First, GC is directly associated with IR. GC enhances the phosphorylations of p38MAPK and c-JNK via the mitogen-activated protein kinase (MAPK) pathway, induces the apoptosis of islet B cells, and destroys glucose sensitivity, thereby promoting gluconeogenesis. Its indirect products, ceramide and diglyceride, can directly affect signal transductions in pancreatic islets through PKC and PI3K pathways (55, 56). GC has also been shown to directly act on nuclear receptors to increase mRNA expressions of hormone-sensitive lipase (HSL) and fatty TG lipase (ATGL) to promote lipolysis (57) and increase concentrations of free FAs acids (FFAs) in the blood, leading to IR. In addition, GC can significantly reduce insulin-stimulated Akt phosphorylation by reducing protein expressions of insulin receptor substrate-1 (IRS-1), PI3K/Akt, and protein kinase B (PKB) (58). It also causes a decrease in expression levels of glucose transporter 1 (GLUT1) protein and reduces the translocation of insulin-stimulated GLUT4 (GLUT4) to the plasma membrane, thereby reducing insulin-stimulated glucose uptake (59), further leading to IR.

Second, the increase in FFA caused by a high level of GC induces the production of CYP2E1, a major enzyme in the microsomal oxidation system. CYP2E1 is the main source of reactive oxygen species (ROS), which is significantly increased in NAFLD patients and is positively correlated with steatosis degree (60, 61). This process may aggravate OS in the mitochondrial environment and further damage organelle functions (62). In addition, excessive release of stress-related corticosteroids can promote peripheral blood leukocyte migration to liver tissues and elevate proinflammatory cytokine levels, such as TNF-α and IL-6. These activated inflammatory cells produce more ROS, thereby exacerbating OS, causing inflammation and liver necrosis (63). Increased ROS activates NF-κB by directly interacting with the small G protein p21ras, and then activating the signaling pathways involved in transforming growth factor-β-activated kinase-1 (TAK1), PI3K, and MAPK 1 (MEK1), which further aggravates liver damage (12).

In hepatocytes, accumulated FFAs act as a danger-associated molecular pattern (DAMP), which produces IL-1β and TNF-α among others by activating the NALP3 inflammasome and inducing lysosomal translocation of Bax (a porin that can trigger lysosomal instability) to promote and maintain inflammation. At the same time, excess FFA can mediate cell apoptosis via various pathways, such as the intrinsic mitochondrial apoptotic pathway, endoplasmic reticulum stress apoptotic pathway, lysosomal apoptotic pathway, and proinflammatory cytokine apoptotic pathway, further aggravating liver damage (62). In addition, under stress conditions, hypothalamus-secreted GC can increase gut permeability (64). Increased gut permeability is closely correlated with NAFLD (see below for details).

#### The Destruction of Gut Microbiota

Human endogenous GM is an important “organ” that provides nutrition, regulates epithelial development, and guides innate immunity. Moreover, it plays an important role in health and disease (65). GM, which is associated with the brain and liver, has recently been correlated with theories of the gut–brain axis (66), gut–liver axis (67), and gut–liver–brain axis (68).

#### Gut Microbiota and Major Depressive Disorder

In MDD animal models, chronic stress affected psychological as well as physiological responses, disrupted the GM (69), and increased gut permeability (70). Overgrowth of gut bacteria, enhanced gut permeability, and bacterial translocation can all lead to elevated lipopolysaccharide (LPS) levels (71–73). Toll-like receptors (TLRs) are pattern recognition receptors for LPS, which are widely present in cell membranes, cytoplasm, and nucleus. TLRs play an important role as bridges in GM-mediated inflammation (74) and are involved in various immune response processes in the body, especially in inducing and promoting inflammation (75). Compared with other TLR-like receptors, activation of TLR4 triggers proinflammatory transcription via 2 adaptor proteins, myeloid differentiation factor 88 (MyD88), and β-interferon-containing TIR domain adaptor protein (TRIF), which induces the activation of NF-κB, AP-1, and IRF3 (76). Activation of these transcription factors causes the release of proinflammatory cytokines, including IL-1β, TNF-α, IL-6, and CXCL10, and elevates cyclooxygenase-2 (COX-2) levels, resulting in activated proinflammatory signals; please refer to the following text in section Release of Inflammatory Factors and references of the details for this content (76). These factors may directly stimulate the HPA axis or inhibit the negative feedback loop to make it upregulated, leading to the release of GC and CRF (77–79). TLRs are expressed in peripheral immune cells and in CNS cells, such as microglia, astrocytes, neurons, and oligodendrocytes (80). Activation of TLR4 can shift microglia polarization toward the M1 phenotype, thereby inducing proinflammatory responses in the CNS (76), which in turn leads to depressive symptoms (80). Astrocytes promote MDD pathophysiology by enhancing serotonin reuptake; however, their potential as TLR-expressing cells in MDD has not been elucidated. Moreover, expressions of TLR4 in astrocytes have not been conclusively determined (80).

IL-1, a GM-produced cytokine in the circulatory system, induces the expressions of COX-2 in cerebrovascular cells (81). It catalyzes the conversion of arachidonic acid (AA) into prostaglandin G2 (PGG2) and further into prostaglandin H2 (PGH2) and then generates prostaglandins (PGs) under the action of cell-specific synthetase (82). Due to their small size and strong lipophilicity, PGs are able to translocate to the brain parenchyma, and their induced synthesis in the brain is necessary for IL-1 to activate the HPA axis (83). At the same time, GM may also exert its effects on the brain through the tryptophan metabolic pathway (84), which may be associated with the gut inflammation state (85–87). When immune activation occurs, inflammatory factors induce the activation of indoleamine-2,3-dioxygenase (IDO), which exists in the periphery. Then, levels of peripheral transient receptor potential (TRP) decrease, and it crosses the blood–brain barrier (BBB) to enter the brain. Meanwhile, the presence of proinflammatory cytokines overactivates the IDO enzyme in the brain, leading to enhanced TRP metabolism, which in turn causes TRP levels in the brain to decrease (88, 89). TRP is converted to 5-hydroxytryptophan (5-HTP) by TPH, which is the rate-limiting step in this pathway. Aromatic amino acid decarboxylase (AAAD) then converts 5-HTP to 5-HT (90). This pathway is closely associated with the occurrence of depression (91, 92).

A high amount of serotonin is localized in the gut, where it is synthesized from tryptophan in the enterochromaffin cells (ECs) of the gastrointestinal tract. It is also present in enteric nerves (84). The vagus nerve can transmit signals to the brain via the paracrine mode (93), thereby triggering depression-like behaviors. However, the specific mechanisms involved in this process remain undefined.

#### Gut Microbiota and Non-alcoholic Fatty Liver Disease

First, GM participates in NAFLD pathogenesis by regulating BA metabolic pathways (94). PXR can be activated by lithocholic acid (LCA), a secondary metabolite of BAs. Transgenic mice expressing a constitutively activated PXR exhibited hepatomegaly and marked hepatic steatosis. Treatment of mice with a PXR agonist elicited a similar effect (95). PXR-induced hepatic steatosis has been attributed to a combination of several mechanisms. First, PXR activation increases FA influx by inducing the expressions of FA translocase (FAT/CD36) in the liver (95), while suppressing FA oxidation by preventing the binding of Forkhead box A2 (FoxA2) to its target genes, such as Cpt1a and Hmgcs2, which are involved in lipid oxidation (96). BA can also bind CAR, an orphan nuclear receptor that is mainly expressed in the liver and small intestines, but its effects contrast with those of PXR. CAR agonist administration significantly suppressed or reversed the adiposity gain induced by carbohydrate-free diet (HFD) feeding in C57BL/6J mice without affecting the lean mass. This reduced adiposity was associated with improved serum lipid profiles and insulin sensitivity (97); however, the specific mechanism has not been established (98). BA can also bind S1P2R. S1P2R-associated activation of neurons led to increased expressions of the proinflammatory chemokine, CCL2, which in turn activated the microglia to secrete more proinflammatory cytokines (99).

After BA stimulation, the L-cell membrane receptor, TGR-5, can activate the accumulation of cytochrome *c* oxidase (COX) and cAMP, increasing the ATP/ADP ratio and cell oxygen consumption. BA promotes glucagon-like peptide-1 (GLP-1) secretion by opening the voltage-gated calcium (CAV) and closing the ATP-sensitive potassium channel (KATP) (100). On the one hand, GLP-1 regulates liver lipid metabolism by enhancing the expressions of FA oxidation genes (acyl-CoA oxidase 1 (AOX1) and peroxisome proliferator-activated receptor alpha (PPARα)) while inhibiting the expressions of sterol regulatory element-binding protein-1c (SREBP-1C) as well as stearoyl–CoA desaturase (SCD1) (101). On the other hand, it may inhibit the release of inflammatory factors (102), reducing endoplasmic reticulum stress (103) and OS (104) to improve IR, inhibit lipid synthesis, promote FA oxidation, reduce liver cell apoptosis, and further improve NAFLD. In the liver, activation of FXR by BAs upregulates the short heterodimer partner (SHP) and PPARα while inhibiting SREBP-1C, thereby suppressing the synthesis of TGs and very-low-density lipoproteins (VLDLs), while enhancing FA oxidation (105, 106).

GM can also affect the BA reabsorption pathway. Most of the conjugated BA (taurocholic acid, glycocholic acid, etc.) is actively reabsorbed by the apical sodium ion-dependent BA transporter on the brush border side of the distal ileum gut epithelial cells (ASBT), combined with ileal BA-binding protein (IBABP), transported to the basement membrane, and reabsorbed by the organic solute transporter (OST) α/β on the basolateral membrane to the portal vein bloodstream, passing through the basolateral hepatocyte sodium ion/sodium taurocholate cotransport peptide (NTCP) uptake on the membrane (107). GM inhibits the expressions of ASBT by stimulating the GATA4 transcription factor (108) and Farnesoid X receptor–SHP–fetoprotein transcription factor (FXR–SHP–FTF) cascade reaction (109), thereby suppressing BA reabsorption. In adipose tissue cells, TGR-5 promotes the conversion of T4 to T3 by upregulating the expressions of type 2 deiodinase (DiO2), thus accelerating metabolic rates in adipose tissues (110).

Second, GM overgrowth, enhanced gut wall permeability, and bacterial translocation all lead to the secretion of large amounts of LPS, which then enters the liver via the portal vein (71–73). Secreted LPS binds LBP in serum, which transmits it to CD14 to form a complex that then causes TLR4 dimerization via MDD-2 and, finally, activates TLR4 (111–113). TLR4 is expressed in various liver cells, such as Kupffer cells, liver cells, hepatic stellate cells (HSCs), bile duct epithelial cells, hepatic sinusoidal endothelial cells, and hepatic dendritic cells. Its activation induces the activation of transcription factors, such as NF-κB, AP-1, and IRF3, leading to the secretion of proinflammatory cytokines, IL-1β, IL-6, and TNF-α, as well as CXCL10, and to the upregulation of COX-2, thereby generating proinflammatory signals and triggering a series of responses. The LPS/TLR4 signaling pathway has been shown to induce inflammation, mediate OS, participate in IR and liver inflammatory damage as well as fibrosis repair, and play a key role in NAFLD pathogenesis (114). Kupffer cells are a type of macrophage that are located in hepatic sinuses and have the ability to phagocytize, process, and present antigens. Due to their special locations, they play an important role in liver defenses. They can effectively phagocytize pathogens entering via the portal vein and act as the first barriers against bacteria and related toxins from the gastrointestinal tract (115, 116). LPS, TLR4, and Kupffer cells play a key role in NAFLD (73, 117). Multiple receptors are expressed on the surface of the plasma membrane of Kupffer cells. LPS binds its receptor, CD14, to activate Kupffer cells to release inflammatory mediators. LPS can also bind TLR4 to activate NF-κB by causing calcium overload in Kupffer cells (118). By stimulating Kupffer cells to activate the TLR4 signaling pathway, LPS induces the secretion of TNF-α, IL-1, IL-6, IL-12, and IL-18 in Kupffer cells (119, 120), thereby triggering hepatic steatosis, IR, and hepatocyte apoptosis, among others.

In addition, GM promotes the release of gastrointestinal hormones through its metabolites (short-chain Fas, SCFAs), thereby regulating IR and insulin sensitivity through its transmembrane receptor G protein-coupled receptor 43 (GPR43) and G protein-coupled receptor 41 (GPR41). These effects induce an increase in intracellular Ca^2+^ and a decrease in cAMP via the G protein pathway Gq/11 and Gi/o (121, 122). GM disorders can also enhance the conversion of choline to toxic methylamine and reduce phosphatidylcholine levels as well as choline bioavailability in the blood. The liver can also metabolize methylamine to trimethylamine *N*-oxide (TMAO), which exposes the host to inflammatory toxic metabolites, resulting in liver manifestations similar to those of a choline-deficient diet (123, 124). Its metabolite, ethanol, can also promote gut permeability and aggravate liver injury (125–127).

### Release of Inflammatory Factors

Inflammatory cytokines, which are elevated in many diseases, are involved in inflammatory responses. After treatment, TNF-α levels in MDD patients have been found to be significantly lower than before (128). Elevated IL-6 levels are significantly positively correlated with psychological stress (129). IL-8 levels have been positively correlated with depressive symptoms and perceived stress, but negatively correlated with social support (130). In a previous study, average plasma IL-18 levels for patients with mental disorders were found to be 3.7 times higher than those of the control group (131). In chronic liver disease, elevated systemic levels of TNF-α are highly correlated with NAFLD severity (132), IL-6 levels are correlated with the degree of liver inflammation (133), and IL-8 levels are significantly correlated with NAFLD disease activity and fibrosis severity (134). Significant correlations between IL-18 and alanine aminotransferase (ALT), gamma glutamyltransferase (GGT), TG, high-sensitivity C-reactive protein (hsCRP), and the degree of liver steatosis have been reported (135). These findings suggest that inflammatory factors in MDD and NAFLD have important mediating roles, and they may be important links between the two conditions. However, the current studies have mainly focused on TNF-α and IL-6, with relatively few studies evaluating IL-8 and IL-18.

#### Inflammatory Factors and Major Depressive Disorder

Currently, there are two types of inflammation hypotheses for MDD. One is that MDD is the product of neurotoxicity and neurodegeneration caused by inflammation, which is mainly related to the tryptophan degradation product (quinolinic acid, QUIN) and kynurenic acid caused by IDO activation. They can destroy post-process components and lead to serious nerve cell degeneration, including the death of hippocampal granular cells and cerebellum meridians selective necrosis (136, 137). Another hypothesis that emphasizes the power factor of stress states that it can micro-damage neurons, resulting in reduced NF release and enhancement of neuroinflammatory activities, and the induction of depressive symptoms (34).

TNF-α can directly stimulate CRF and overactivate the HPA axis, which in turn leads to the secretion of large amounts of ACTH and GC (138). Besides, it prevents the cortisol–GR complex from entering the nucleus by activating the JNK signaling pathway. It also induces the NF-κB signaling pathway to prevent the complex from binding to DNA, causing GR resistance, expression decrease, or functional decrease (139), which induces depression. Meanwhile, TNF-α affects CRF activity of the central amygdala, increases the uptake of 5-HT and NE in PVN (140), and causes the degradation of the 5-HT synthetic raw material-TRP by stimulating IDO (141, 142). At the same time, levels of the degradation product (QUIN) increase, which in turn suppresses NF while enhancing neurotoxicity (143). Moreover, it induces depression by activating the serotonin transporter (SERT) through the p38/MAPK pathway and by suppressing 5-HT (144). TNF-α affects synaptic plasticity and neuronal atrophy by inhibiting negative feedback regulation of the HPA axis through the JNK, P38, and NF-κB signaling pathways. However, the specific mechanisms related to apoptosis, survival, and MDD induced by these pathways and relationships with TNF-α concentrations should be evaluated further (145–147).

Since TNF-α and interferon-γ (IFN-γ) as well as other inflammatory factors can induce the synthesis and secretion of IL-6 (148), IL-6 can play a similar role to TNF-α by excessively activating the HPA axis (138) and stimulating IDO activation to degrade tryptophan and hinder the synthesis of *N*-acetyl-5-HT and melatonin (148). Detailed mechanisms have been previously described (149, 150). IL-6 can play both anti-inflammatory and proinflammatory functions. It exerts its anti-inflammatory effects by binding receptors on cell membrane surfaces. This process is referred to as classical signal transduction, but it only occurs on certain subsets of T cells, hepatocytes, megakaryocytes, neutrophils, and monocytes. When the soluble form of IL-6 receptor (sIL-6R) is detached from membrane-bound receptors and binds IL-6, it can be transported to any type of cells expressing membrane-bound glycoprotein 130 (gp130) to produce proinflammatory signals. This process is referred to as anti-signal transduction. In these two signal transduction processes, the IL-6/IL-6R/gp130 complex activates intracellular signal transduction through the JAK/STAT pathway and the MAPK pathway (151).

#### Inflammatory Factors and Non-alcoholic Fatty Liver Disease

TNF-α is highly correlated with IR. It was the first proinflammatory factor to be associated with IR (152). It can inhibit IRS-1 tyrosine phosphorylation and downstream signal transduction by suppressing GKAP42 levels (153) and further inhibit GLUT4 translocation (154, 155), thereby inducing IR. It also induces the expression of Suppressor of Cytokine Signal Transduction 3 (SOCS3) to inhibit its downstream signals, JAK and STAT3, leading to IR (156, 157). In addition, it can damage adiponectin multimerization and, consequently, decrease adiponectin secretion by altering disulfide bond modification in the endoplasmic reticulum and increasing the sensitivity of hepatocytes to insulin (158).

Second, in addition to causing IR, TNF-α can also promote cell damage and apoptosis. After TNF-α binds its receptor (TNFR), it interacts with tumor necrosis factor receptor-related protein and death domain (TRADD), tumor necrosis factor receptor-related factor 2 (TRAF2), and receptor-interacting protein (RIPK). Together, they form TNF-α-induced signal complex I (complex I) and activate the NF-κB signaling pathway to form a proinflammatory and anti-apoptotic pathway (147), which may be affected by the IκK complex, pyruvate dehydrogenase kinase 4 (PDK4), and regulation of p65 to promote apoptosis (159, 160). Besides, if TRADD, TRAF2, and RIPK are separated from their receptors, they recruit fas-related death domain protein (FADD) and precaspase-8 (Caspase 8) to form a TNF-α-induced signal complex II (complex II), which in turn binds ROS regulatory factor 1 (Romo1) located in the mitochondria and recruits Bcl-xl protein to reduce the mitochondrial membrane potential and to activate ROS as well as JNK to promote cell apoptosis (147, 159). The mechanisms involved in the activation of the JNK signaling pathway and NF-κB pathway have been previously described (161).

In addition, TNF-α can also induce liver steatosis by inhibiting the activity of AMP-activated protein kinase (AMPK), activating SREBP-1c, and upregulating the expression of acetyl-Coenzyme A carboxylase (ACC) as well as FA synthase (FAS). These effects lead to increased FA synthesis and excessive accumulation of TG, which results in liver steatosis and direct induction of lipid accumulation in HepG2 cells (162, 163).

As mentioned earlier, TNF-α can induce the production and secretion of IL-6, which may associate with TNF-α to cause diseases and aggravate NAFLD (163). IL-6 has also been associated with phosphorylation of STAT3, ERK, and JNK to indirectly induce changes in AKT and mTOR-S6K signals. Suppressed IL-6 inhibits the phosphorylations of STAT3, ERK, and JNK and reverses the decline in p38 phosphorylation (164). Activated JNK, STAT3, ERK, and AKT signaling pathways are involved in IR and apoptosis (165–167). However, IL-6 has also been shown to play protective roles in chronic liver disease, and blocking IL-6 signaling can improve IR as well as liver damage (168, 169). These contradictory outcomes may be associated with the two contrasting pathways involved in IL-6-mediated anti-inflammatory and proinflammatory effects. However, studies should aim at elucidating these outcomes.

### Changes in Thyroid Hormone Levels

#### Thyroid Hormones and Major Depressive Disorder

Alterations in the HPA axis and hypothalamic–pituitary–thyroid (HPT) axis are important neuroendocrine abnormalities in MDD. However, their associated mechanisms have not been established, and there is no unified theory on the relationship between MDD and thyroid hormone (TH) levels (170).

Most studies report that the prevalence of subclinical hypothyroidism (SCH) and obvious hypothyroidism in MDD patients is higher than that of the general population. About 4–40% of patients with affective disorders have SCH (171, 172), and the prevalence of SCH in female MDD patients is about twice that of males (173). However, some studies have reported contrasting conclusions to the effect that there is no correlation between SCH and depression (174, 175). Other studies have documented that the prevalence and incidence of hypothyroidism or hyperthyroidism in patients with severe MDD are higher than those of the general population (176). In addition, contradictory conclusions have been reported with regard to other aspects. For instance, some studies claim that the correlation between MDD and SCH risk is more obvious in people aged over 50 years (172), while another study reported that it is associated with younger patients (<60 years old), but not elderly patients (177).

Geography (178), diet (179), personal habits (180), depression scale scores, thyroid-stimulating hormone (TSH) ranges, and other indicators do not have a unified standard, which may lead to differences in results. Since 17.6% to 30% of patients with overt thyroid disease were reported to have SCH due to inadequate therapy, SCH or mild thyroid dysfunction in patients with MDD should be paid more attention to in clinical practice (171).

#### Thyroid Hormones and Non-alcoholic Fatty Liver Disease

Hypothyroidism is closely associated with NAFLD (128, 181, 182). Hypothyroidism is a metabolic disease that is characterized by decreased serum TH and increased TSH levels (183), which may be related to lipid metabolism, OS, and mitochondrial dysfunction. Moreover, it mediates NAFLD occurrence and development.

TSH can bind TSH receptor (TSHR) on the surface of liver cells, mediate peripheral FFA into the cell through FA-binding proteins (L-FABPs), FA transfer proteins (FATPs), and FA translocators (FAT), and increase liver SREBP-1c activities through the cAMP/PKA/AMPK pathway. TSH has also been shown to increase liver gluconeogenesis and TG accumulation, inhibit BA synthesis, and cause high cholesterolemia by regulating the phosphorylation of HMG-CoA reductase (a cholesterol synthesis rate-limiting enzyme) and the activity of PPAR-α, thereby leading to NAFLD occurrence (184–187). The remaining FAs are released into the blood in the form of VLDLs and hydrolyzed by lipoprotein lipase (LPL) to become low-density lipoproteins (LDLs). LDL binds LDLR receptor to cell membranes and returns to the cytoplasm (188). TH can increase the expressions and enzyme activities of LPL. Suppressed TG clearance leads to accumulation of TG in the liver when peripheral blood TH decreases, which in turn leads to NAFLD (VLDL is rich in TG cholesterol, etc.) (188). In addition, TH promotes FA catabolism through liver lipid phagocytosis (autophagy of FAs in liver cells), transports lipids to lysosomes, and stimulates β-oxidation (189). Other hormones such as leptin, adiponectin, fibroblast growth factor-21, and sex hormones are all related to TH (188, 190).

### Effects of Obesity

Obesity is one of the most important public health challenges of the 21st century, reaching epidemic proportions. It affects adults, adolescents, and children of both genders (191). In addition to the established medical comorbidities, obesity is also associated with psychiatric challenges and the developments of many diseases, including cardiovascular diseases, diabetes, obstructive sleep apnea, NASH, osteoarthritis, and mobility-related disorders (192). Nevertheless, the pathogeneses for these diseases have not been fully established because these proximal mechanisms may be seriously affected by more distal behavioral and psychosocial factors (193).

#### Obesity and Major Depressive Disorder

With various studies establishing a link between obesity and MDD, these conditions are bidirectional. The presence of one increases the risk of developing the other. This may be related to genetics, alterations in systems involved in homeostatic adjustments (HPA axis, immuno-inflammatory activation, and neuroendocrine regulators of energy metabolism such as leptin, insulin, and microbiome), and brain circuitries integrating homeostatic and mood regulatory responses (193).

Long-term HPA axis hyperactivation has also been found in nearly half of adult obese persons (194). At a young age, an almost 10-fold increased risk of obesity has been observed in children with the highest long-term cortisol levels (195). This is a result of chronic inflammation that is typical of obesity, which may disrupt GR functioning. Proinflammatory cytokines activate elements of the cellular transduction cascades that impede GR nuclear translocation or interfere in GR interactions with response elements in gene promoters (196).

Chronic low-grade inflammation is a hallmark of obesity. White adipocytes infiltrated by macrophages and other immune cells produce proinflammatory cytokines (197). This peripheral immune activation can be translated via humoral, neural, and cellular pathways (198) into brain inflammation, as indicated by higher hippocampal and cortical expressions of cytokines in obesity animal models (199, 200).

The effects of leptin on moods may be exerted via different pathways: direct action on neurons via receptors expressed in the hippocampus and amygdala, enhancement of neurogenesis and neuroplasticity in the hippocampus and cortex, and modulation of the HPA axis and immune systems (201, 202). It has been hypothesized that leptin resistance (peripheral hyperleptinemia due to reduced central signaling) may constitute a phenotype risk of depression (203). Alterations in regional cerebral metabolism due to IR have been associated with memory and executive function impairments as well as neuronal damage in the hippocampus and medial prefrontal cortex (204, 205). Therefore, it has been postulated that insulin dysregulation plays a role in neuropsychiatric conditions such as dementia and depression (206).

Microbiota of the gastrointestinal tract is emerging as a key player in the pathophysiology of obesity. Alterations in microbiota are markers of local inflammation, which can increase gut permeability to bacteria that, in turn, contributes to the onset and progression of systemic inflammation.

#### Obesity and Non-alcoholic Fatty Liver Disease

Obesity is a primary antecedent to NAFLD, which makes them closely related (207). Up to 80% of NAFLD patients are obese, and only 16% of individuals with a normal body mass index (BMI) and without metabolic risk factors are obese (208, 209). Obesity is aggravated by lipid metabolism, systemic inflammation, intestinal microorganisms, and IR.

Fat accumulation in the liver is the first step in NAFLD development. The higher the visceral fat content, the greater the risk of metabolic abnormalities. Visceral adipose tissue (VAT) has an abundant vascular and neural distribution than in other parts, which makes its cell metabolism more active, sensitive to sympathetic nervous system regulation, and produce more FFAs (210). VAT is mostly found in mature and hypertrophic adipocytes. Compared with the small adipocytes, their FFA and TG uptake rates are low, which makes it easy to cause fat deposition (211).

Excess FFAs and chronic low-grade inflammation from VAT are two of the most important factors contributing to liver injury progression in NAFLD. In hepatocytes, FFAs are bound to coenzyme A as fatty acyl-CoAs to form hepatic TGs, which stimulate the reduction of insulin-induced glucose uptake as well as induce intracellular inflammation and IR (212, 213). The hyperinsulinemia and hyperglycemia that occur with IR create an imbalance in lipid input relative to output and promote hepatic steatosis (214).

The associations between GM and the pathogenesis of obesity as well as NAFLD have been reported (215–217). One mechanism is that colonic-derived SCFAs account for 10% of harvested energy from the diet, with acetate (being mainly obesogenic) being the main source of energy (218). Besides, acetate is the most substantially absorbed SCFA and is a substrate for lipogenesis and cholesterol synthesis in the liver and adipose tissue (219). GM can also decrease intestinal expressions of the adipose tissue LPL inhibitor fasting-induced adipose factor (FIAF). The net result is increased uptake of FAs in the adipose tissues and liver, favoring the expansion of the adipose tissue and hepatic steatosis (215).

In addition, GM contributes to the development and progression of NAFLD through several mechanisms: modulation of intestinal permeability by promoting endotoxemia as well as other microbe products that promote systemic and hepatic inflammation; modulation of choline metabolism (required for VLDL synthesis and export of lipids from the liver); generation of endogenous ethanol as well as other toxic products such as toxic compound TMAO; and modulation of BA homeostasis and FXR functions (220).

## Conclusions

There is a significant correlation between MDD and NAFLD, and they mediate and promote each other, gradually forming a vicious circle, which is particularly obvious in patients with MDD-associated NAFLD. The most common biological feature for MDD patients is that long-term life pressure and psychological stress chronically elevated GC levels (33). Elevated GC can directly induce MDD; for another, the indirect products of inflammatory factors increase, and GM disturbance further promotes and aggravates MDD and NAFLD.

**Figure 1:**
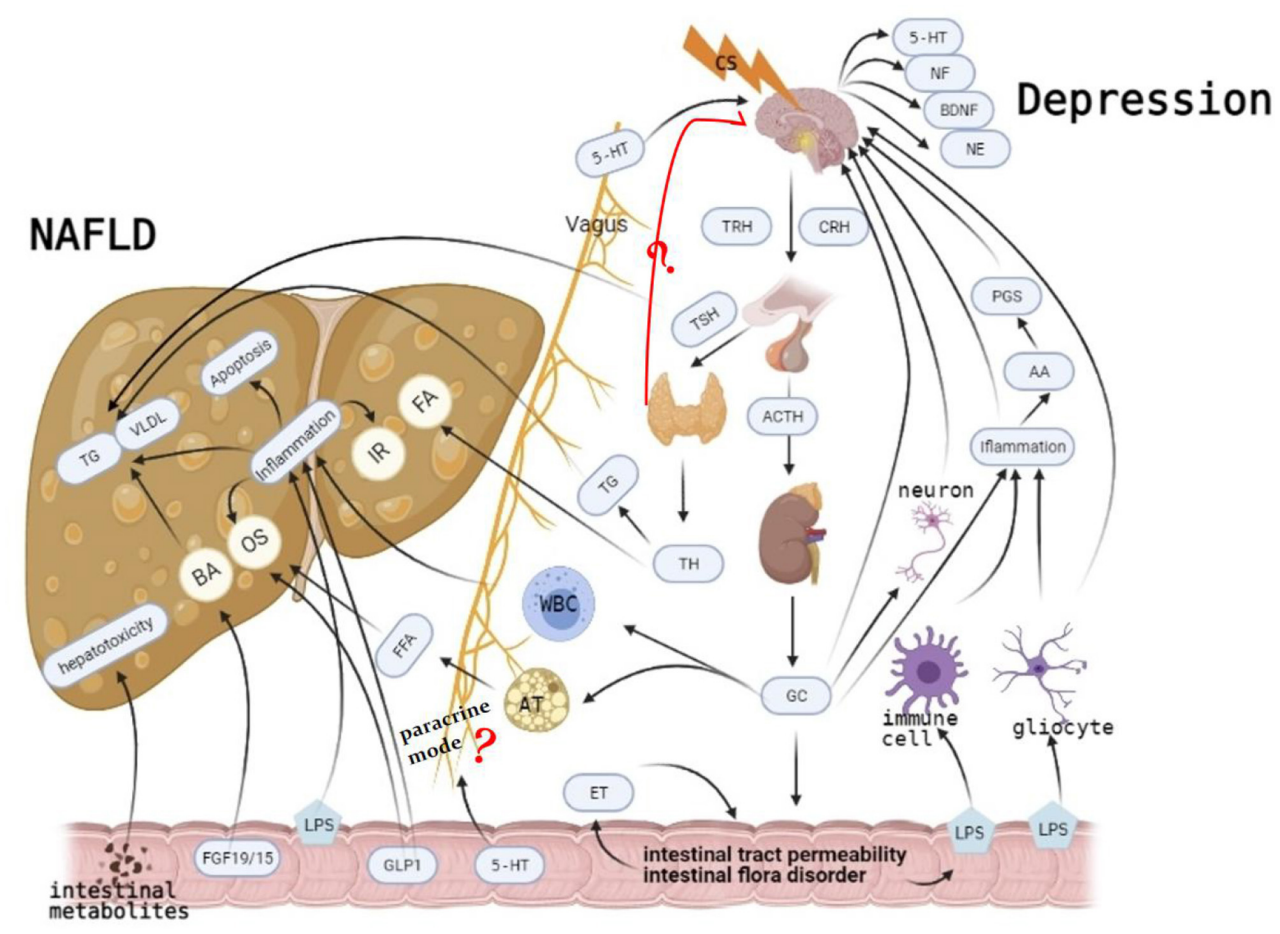
Long-term psychological stress induces the increase and accumulation of glucocorticoid (GC). On the one hand, it induces non-alcoholic fatty liver disease (NAFLD) by mediating liver transport of white blood cells and decomposition of fat cells. It also mediates major depressive disorder (MDD) occurrence by elevating inflammatory factors, damaging neurons, and inhibiting the negative feedback of the hypothalamic–pituitary–adrenal (HPA) axis. It also affects intestinal permeability and intestine flora, gradually leading to mutual promotions of NAFLD and MDD, causing disease outbreaks. Although more studies have shown that hypothyroidism is more related to MDD, there is still a big divergence between thyroid function and MDD. Its specific mechanism, which causes MD through paracrine mode, is still unclear. Similarly, the specific mechanism of MDD caused by intestinal flora through paracrine is not clear. Causal relationships between them should be further studied.

Therefore, we postulate that chronic elevations in GC levels may be key in the pathogenesis of MDD-related NAFLD. Long-term psychological stress can induce the increase and accumulation of GC. If not controlled, when GC concentrations reach a certain level, they can induce the release of inflammatory factors and change intestinal permeability (63). Elevated inflammatory cytokines activate the HPA axis (86), which elevates GC levels. Meanwhile, changes in gut permeability further promote the release of inflammatory factors (80), causing disease outbreaks. Changes in TH induce MDD and NAFLD, although interaction mechanisms and causality between GC and TH should be further studied (Figure 1).

Not only is MDD related to NAFLD, but it is also associated with metabolic diseases such as diabetes. Since the concept of “microbe–gut–brain axis” was first put forward in 2012 (221), studies have begun to pay attention to the relationship between intestinal microbes and the brain. Mental disorders can lead to abnormal glucose metabolism and increase the risk of diabetes. However, diabetes can also directly or indirectly affect the function of the CNS, leading to the emergence of mental symptoms. Interactions between nervous system diseases and metabolic diseases increase the risk of complications and early death (222). This process may be highly related to the HPA axis (223, 224), monoamine neurotransmitters (225, 226), immune inflammation (227), cellular signaling pathway (228, 229), and the metabolic pathway (230, 231), among others. However, the specific mechanisms of action between the two systemic diseases have not been clarified.

Currently, there is no good management strategy for the cross talk between the two diseases. For example, the use of psychotropic drugs, including antidepressants, lithium salts, and antipsychotics, is associated with a higher prevalence of diabetes, among which clozapine, olanzapine, and quetiapine are correlated with the highest risks (232). If the patient has increased blood glucose levels, other antipsychotic drugs should be considered. In recent years, the integration of psychiatric nursing in diabetes education clinics has been considered as an effective management option for diabetes mellitus with depression (233). However, this mode of management needs further exploration.

In MDD-related NAFLD, we should prevent and treat from the source by improving our lifestyles and reducing our stress. Aerobic exercise is an effective way of stress reduction (234). People who exercise regularly have a higher level of mental health. Physical exercise can reduce work pressure and improve job satisfaction and happiness (235). Aerobic exercise has been shown to significantly improve serum ALT, aspartate aminotransferase (AST), and NAFLD activity scores in NAFLD patients and has a certain effect on TG (236, 237). In early and middle stages of the disease, GC inhibitors or antagonists may be key for the treatment of NAFLD. A few studies have investigated this perspective, although treatment of NAFLD from the perspective of GC has achieved significant effects (238). In the middle and late stages of the disease, GC, inflammatory factors, GM, and TH, among others, interact in a vicious circle. Combination medication may be an optional treatment. However, combined medications are associated with significant side effects. To reduce these risks, there is a need to ascertain the causal relationship between them as well as the dosage and order of medication.

The development of traditional Chinese medicine (TCM) has made it possible to treat complex diseases. In the TCM theory, the function of “liver governing free flow of qi” is an important theory for maintaining MDD and NAFLD, which involves the regulation of emotional response (nervous system) and digestive function (digestive system). The liver regulates the free flow of qi and prefers to act freely and dislikes depression, which will be destroyed by emotional dysfunction and cause qi stagnation, gradually leading to MDD. If liver qi stagnates for a long time, it induces liver inflammation, which leads to liver damage and then causes NAFLD development. In clinical medication, MDD and NAFLD can be treated with Sini powder (a prescription in TCM), which has been verified by relevant experiments and has achieved good therapeutic effects (239, 240). These outcomes may be highly related to its multi-target and multi-path characteristics (241–243). However, due to the complexities of TCM compounds and the possibility of chemical composition changes after processing (244), studies on Chinese medicine are challenging. We believe that this challenge will be easily solved with advances in science and technology.

## Data Availability Statement

The original contributions presented in the study are included in the article/supplementary material, further inquiries can be directed to the corresponding author/s.

## Author Contributions

QS and YW: writing—original draft preparation. QS, TX, and QY: writing—article revision. CM, FC, and XW: writing—review and editing. XL and JJ: drawing—editing of picture. All authors have read and agreed to the published version of the manuscript.

## Funding

This research was funded by the General Program of the National Natural Science Foundation of China (Nos. 81774122 and 82004327) and Key project of Beijing University of Chinese Medicine (No. 2020-JYB-ZDGG-004).

## Conflict of Interest

The authors declare that the research was conducted in the absence of any commercial or financial relationships that could be construed as a potential conflict of interest.

## Publisher's Note

All claims expressed in this article are solely those of the authors and do not necessarily represent those of their affiliated organizations, or those of the publisher, the editors and the reviewers. Any product that may be evaluated in this article, or claim that may be made by its manufacturer, is not guaranteed or endorsed by the publisher.
